# Aptamer-Based Western Blot for Selective Protein Recognition

**DOI:** 10.3389/fchem.2020.570528

**Published:** 2020-10-29

**Authors:** Yao Wang, Zhe Li, Hanyang Yu

**Affiliations:** ^1^Department of Biomedical Engineering, College of Engineering and Applied Sciences, Jiangsu Key Laboratory of Artificial Functional Materials, Nanjing University, Nanjing, China; ^2^State Key Laboratory of Coordination Chemistry, Nanjing University, Nanjing, China; ^3^Chemistry and Biomedicine Innovation Center (ChemBIC), Nanjing University, Nanjing, China; ^4^State Key Laboratory of Analytical Chemistry for Life Science, Nanjing University, Nanjing, China

**Keywords:** aptamer, Western blot, *in vitro* selection, protein recognition, functional nucleic acid

## Abstract

Selective protein recognition is critical in molecular biology techniques such as Western blotting and ELISA. Successful detection of the target proteins in these methods relies on the specific interaction of the antibodies, which often bring a high production cost and require a long incubation time. Aptamers represent an alternative class of simple and affordable affinity reagents for protein recognition, and replacing antibodies with aptamers in Western blotting would potentially be more time- and cost-effective. In this work, multiple fluorescent DNA aptamers were isolated by *in vitro* selection to selectively label commonly used tag proteins including GST, MBP, and His-tag. The generated aptamers G1, M1, and H1 specifically bound to their cognate target proteins with nanomolar affinities, respectively. Compared with conventional antibody-based immunoblotting, such aptamer-based procedure gave a cleaner background and was able to selectively label target protein in a complex mixture. Lastly, the identified aptamers were also effective in recognition of different fusion proteins with the same tag, thus greatly expanding the scope of the potential applications of these aptamers. This work provided aptamers as useful molecular tools for selective protein recognition in Western blotting analysis.

## Introduction

Western blotting is a standard molecular biology technique for sensitive and selective protein detection (Nybo, 2012; Kim, 2017). The common procedure of Western blotting analysis starts with electrophoresis separation of proteins followed by transfer to a nitrocellulose membrane. After blocking for non-specific adsorption, specific protein recognition is achieved by interaction with an appropriate primary antibody and secondary antibody, which is often conjugated to a fluorophore or an enzyme such as horseradish peroxidase (Mishra et al., 2019; Han et al., 2020). Although Western blotting is very useful for protein identification and quantification, the whole procedure is laborious and time-consuming. In addition, antibodies as the key affinity reagent usually suffer from high production cost and are prone to irreversible denaturation (Groff et al., 2015). Therefore, to develop a simpler, more robust and affordable Western blotting technique that does not rely on antibodies would be useful to biomedical researchers.

Aptamers are short single-stranded oligonucleotides that bind their targets with high affinity and specificity by forming distinct tertiary structures (Ellington and Szostak, 1990; Robertson and Joyce, 1990; Tuerk and Gold, 1990; Wang et al., 2019). Aptamers have been generated by *in vitro* selection to recognize a wide variety of targets ranging from metal ions, small organic compounds and biomacromolecules (Tuleuova et al., 2010; Sharma et al., 2012; Qu et al., 2016; Ruscito and DeRosa, 2016) to viruses, bacteria and human cells (Medley et al., 2011; Wandtke et al., 2015; Marton et al., 2016; Wang et al., 2018). Compared with antibodies, aptamers possess several advantageous features as protein affinity reagents (Groff et al., 2015; Crivianu-Gaita and Thompson, 2016; Kooshki et al., 2019). First, aptamers can be generated to bind protein targets that are poorly immunogenic or toxic to animals. Moreover, aptamers can refold into the native structures and restore their functions after thermal denaturation, while the denaturation process of an antibody is irreversible. Lastly, once identified, aptamer sequences can be chemically synthesized and modified on a large scale, thus greatly reducing the production cost.

Utilizing aptamers as affinity reagents in specific protein blotting analysis has been sometimes referred to as aptablotting or South-Western blot (Li et al., 2017; Sekhon et al., 2017). However, in most cases only the primary antibody was replaced by an aptamer (Drolet et al., 1996; Murphy et al., 2003; Tsuji et al., 2009; Liu et al., 2012; Martin et al., 2013; Woo et al., 2015; Frezza et al., 2020). And therefore, a second incubation and further color development reaction was required. Additionally, these procedures often required overnight blocking or incubation, so these methods do not compare favorably with conventional Western blot in terms of simplifying the protocol and saving time. Alternatively, aptamers could be directly attached to a reporter moiety such as fluorophore and quantum dot, which would make protein detection more straightforward. Indeed, Western blot techniques using aptamer-functionalized quantum dots or gold nanoparticles have been reported (Shin et al., 2010; Li et al., 2011). However, these methods required an additional conjugation step, and the results suffered from very high background fluorescence, or involved a complementary strand to reduce non-specific adsorption. Therefore, it is highly desirable to develop a simple, selective, and robust aptamer-based protein blot technique.

In this work, we selected DNA aptamers toward several commonly used tag proteins, and developed an aptamer-based Western blotting technique, which requires fewer steps and reduces non-specific binding. First, to generate aptamers suitable for such applications, three *in vitro* selection experiments of aptamers toward glutathione S-transferase (GST), maltose-binding protein (MBP), and poly(histidine) tag (His-tag) proteins were performed. The isolated aptamers bound selectively to the cognate target proteins immobilized on nitrocellulose membrane with nanomolar affinities. When compared with conventional antibody-based Western blotting, aptamer-based protein recognition assay was able to selectively label the target protein from a complex mixture and gave a cleaner background. Lastly, the aptamers were also capable of recognizing fusion proteins carrying these tags, thus greatly expanding the scope of potential applications of such aptamers. This work provided aptamers as useful molecular tools for selective protein recognition in Western blot analysis.

## Materials and Methods

### Materials

All the chemicals were purchased from Aladdin (Shanghai, China) and Sangon Biotech (Shanghai, China). All the buffer solutions were prepared with Millipore water. Target proteins including glutathione S-transferase (GST), maltose-binding protein (MBP), poly(histidine)-tagged green fluorescent protein (His-GFP), and poly(histidine)-tagged thioredoxin 1 (His-TrxA) were expressed in *E. coli* and purified by affinity chromatography. If not stated otherwise, all the DNA sequences were purchased from Sangon Biotech (Shanghai, China), dissolved in ddH_2_O and quantified on NanoDrop. Anti-GST antibody was purchased from Zoman Biotechnology (Beijing, China).

### Protein Expression and Purification

The plasmids encoding His-GFP, His-TrxA, GST, MBP, GST-GFP, GST-TrxA, MBP-TvaF (a cysteine decarboxylase), and MBP-RPA2 (replication protein A2) proteins were constructed by homologous recombination. Transformation of *E. coli* BL21 (DE3) competent cells were carried out according to the manufacturer's protocol. Briefly, 100 ng of the plasmids were added to 50 μL *E. coli* competent cells and incubated for 30 min on ice. The mixture was heated at 42°C for 45 s. and then cooled on ice for 5 min. Five hundred microliter of LB medium (10 g/L NaCl, 10 g/L tryptone, and 5 g/L yeast extract) were added and cultured for 1 h in a 37°C shaker. Cells containing the plasmids were plated on agar plates containing appropriate antibiotics. The plates were incubated at 37°C overnight and clones were picked from plates for further culture and analysis.

*E. coli* cell cultures in 300 mL LB medium containing 50 μg/mL antibiotic were used for protein expression. When OD_600_ reached 0.7, target protein expression was induced by adding 0.2 mM IPTG (isopropyl β-D-thiogalactoside) into the flask. The culture was grown for another 6 h at 37°C, and the cells were harvested by centrifugation at 8,000 rpm at 4°C for 10 min. Following washing with cool PBS (KH_2_PO_4_ 2 mM, Na_2_HPO_4_ 8 mM, NaCl 136 mM, KCl 2.6 mM, pH 7.4), cell lysis with ultrasonic homogenizer and centrifugation, the supernatants were loaded onto appropriate affinity columns (Ni-NTA column for His-tagged proteins; glutathione agarose resin column for GST protein; amylose resin column for MBP). Lastly, the target protein was washed using 5 mL wash buffer (20 mM Tris-HCl, pH 7.9, 0.5 M NaCl and 20 mM imidazole for His-tagged proteins; PBS for GST protein; 20 mM Tris-HCl, pH 7.4, 0.2 M NaCl, and 1 mM EDTA for MBP) twice, and eluted from the affinity columns using a 5 mL elution buffer (20 mM Tris-HCl, pH 7.9, 0.5 M NaCl and 100 mM imidazole for His-tagged proteins; PBS containing 10 mM reduced glutathione for GST protein; 10 mM maltose, 20 mM Tris-HCl, pH 7.4, 0.2 M NaCl for MBP). The purified proteins were stored at −20°C.

### *In vitro* Selection

The initial DNA library design contains a central 40-nucleotide (nt) randomized region flanked by an upstream 19-nt and a downstream 18-nt fixed sequence regions. This single-stranded DNA (ssDNA) library was prepared by solid-phase synthesis and purified on denaturing PAGE. In each round of selection, the purified target proteins (GST, MBP, His-GFP, and His-TrxA) were first electrophoresed in 12% SDS-PAGE and transferred to a nitrocellulose (NC) membrane. Subsequently, the NC membrane was blocked by incubating with 1.9 mL binding buffer (20 mM Tris-HCl, 100 mM NaCl, 5 mM MgCl_2_, and 0.1% bovine serum albumin) supplemented with 10 μg/mL yeast tRNA and 10 μg/mL salmon sperm DNA at room temperature for 15 min. One nanomole of initial fluorescently-labeled ssDNA library, dissolved in 100 μL binding buffer, was first allowed to fold by heating at 95°C for 5 min and cooling at room temperature for 10 min. Folded ssDNA library was added to NC membrane with immobilized target protein and incubated for 1 h. After incubation, the NC membrane was washed with binding buffer containing NaCl of increasing concentrations four times and scanned with LI-COR Odyssey infrared imaging system (Ex: 675 nm, Em: 694 nm). The band corresponding to aptamer-protein complex was excised, and ssDNA was eluted using 100 μL ddH_2_O by heating at 95°C for 10 min. The collected ssDNA was amplified by PCR using Cy5.5-labeled forward primer and biotin-labeled reverse primer. The PCR program includes: 95°C for 5 min; 95°C for 15 s, 52°C for 30 s, 72°C for 30 s, 25 cycles; 72°C for 10 min. The amplified dsDNA product was captured on a streptavidin-coated resin. The Cy5.5-labeled strand was separated from biotin-labeled strand by incubating in 100 μL NaOH (200 mM) at room temperature for 2 min, 3 times. This enriched ssDNA pool was used to initiate the next round of selection. During the process, selection stringency was incrementally increased by (i) decreasing target protein concentration from 3.5 to 1.0 mg/mL; (ii) increasing blocking yeast tRNA and salmon sperm DNA concentration from 10 to 500 μg/mL; (iii) increasing NaCl concentration in binding buffer in washing step from 150 to 500 mM; (iv) changing washing conditions from 5 min for 4 times to 10 min for 6 times. After 18 rounds of *in vitro* selection and amplification, the enriched DNA pool was cloned into pEasy-T1 vector and sequenced. The sequencing results were analyzed and compared with previously reported aptamer sequences using Clustalx sequence alignment.

### Dot Blot Assay

Aptamers were diluted to 50 nM with the binding buffer, followed by heating for 5 min at 95°C and cooling on ice for 10 min. Target proteins were prepared as a series of 2-fold dilutions, starting from 1 or 2 μM. Negative control was aptamer alone, without any proteins.

Nitrocellulose membrane was pre-treated with 0.4 M fresh KOH solution for 30 min and washed twice by ddH_2_O. Subsequently, all three membranes (NC, nylon, and filter paper) were equilibrated in binding buffer for 30 min at 4°C. The membranes were assembled into a dot blot apparatus in the order of nitrocellulose, nylon, and filter paper from top to bottom. One hundred microliter solution of aptamer-protein mixture was added to each well, allowing unbound aptamer pass through nitrocellulose while retaining aptamer-protein complex on nitrocellulose membrane. The membranes were washed again using 200 μL binding buffer. The blots were imaged and analyzed with an Odyssey infrared imaging system.

### Gel Shift Assay

Cy5.5-labeled aptamers were firstly heated at 95°C for 5 min and cooling on ice for 5 min. Target proteins were prepared as a series of 2-fold concentration dilution, starting from 16 μM. Fifty nanomolar aptamers were allowed to interact with target proteins in the binding buffer (50 mM Tirs-HCl, 100 mM NaCl and 5 mM MgCl_2_, pH 7.5) at room temperature for 1 h. Three microliter loading buffer (30% glycerin) was added and the samples were separated on a 8% native polyacrylamide gel (19:1 acrylamide:bisacrylamide) at 100 V until the blue dye marker run to the bottom of gel. Finally, the gel was imaged and analyzed with an Odyssey infrared imaging system.

### Western Blot

Purified proteins and cell lysates were denatured in loading buffer by heating for 10 min at 99°C. One microgram of proteins were loaded and separated by 12% SDS-PAGE and transferred to the NC membrane.

In aptamer-based protein blotting, the membrane was first blocked with 100 μg/mL yeast tRNA and 100 μg/mL salmon sperm DNA in binding buffer for 15 min at room temperature. The membrane was then incubated with a Cy5.5-modified aptamer for 1 h. After washing, the membrane was imaged with an Odyssey infrared imaging System.

In the conventional antibody-based Western blot, the membrane was first blocked with 2% BSA for 1 h at room temperature. The membrane was then probed with anti-GST rabbit polyclonal antibody for 2 h. After washing, the membrane was incubated with a Dylight 800 modified secondary antibody for 1 h in the dark. Lastly, the membrane was imaged with an Odyssey infrared imaging system.

## Results

**Scheme 1:**
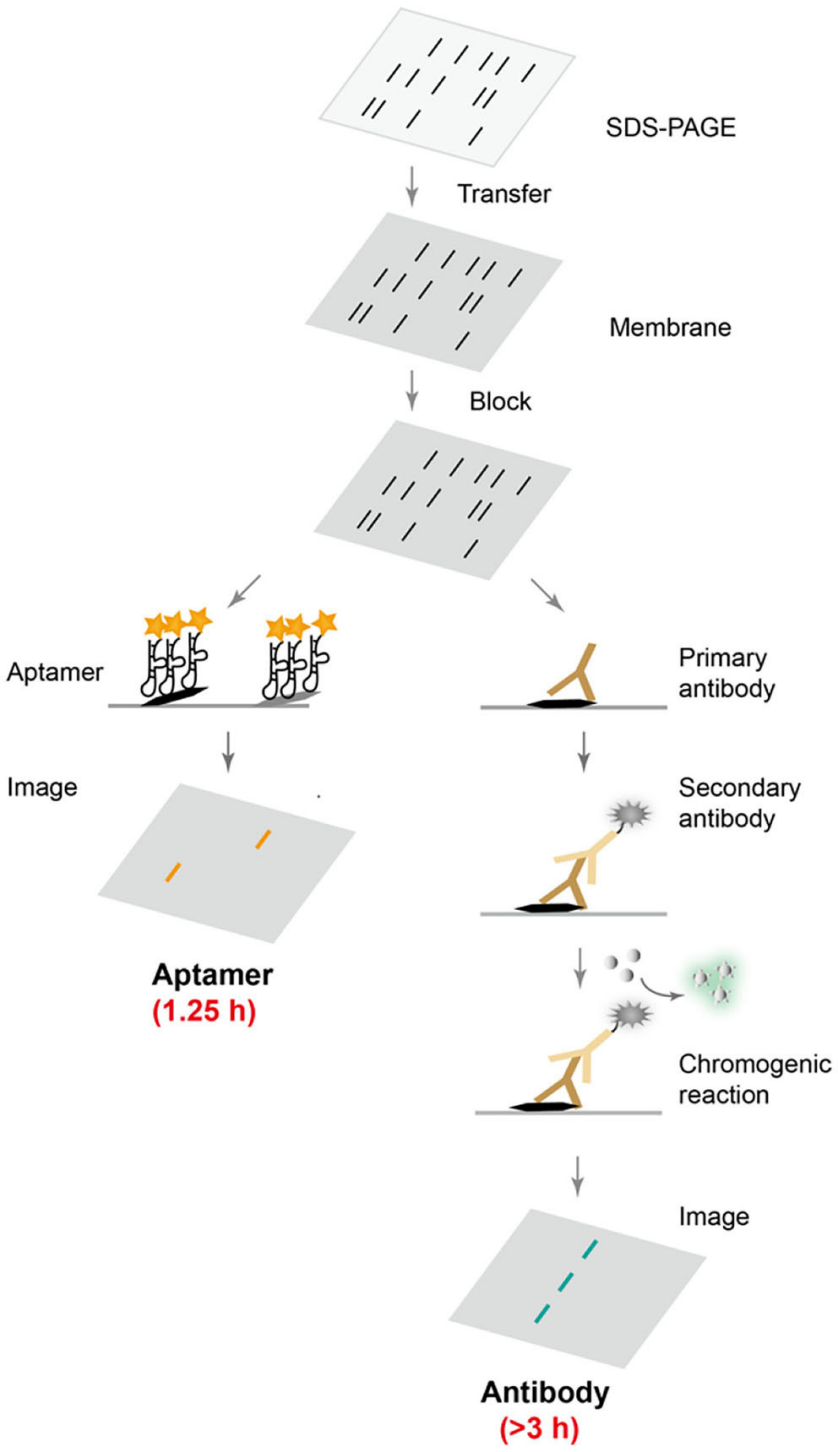
Comparison of aptamer- and antibody-based protein blotting analysis. After common electrophoresis, transfer and blocking steps, fluorescently labeled aptamers can be directly applied for selective protein recognition, while conventional Western blot requires two incubation steps with primary and secondary antibodies, followed by a chromogenic reaction and chemiluminescence detection.

Although antibodies are the most widely used protein affinity reagents in techniques such as Western blotting and ELISA, antibodies suffer from high production costs and batch-to-batch variations, and often require long incubation with target proteins to achieve optimal binding interactions (Uhlen et al., 2016; Pillai-Kastoori et al., 2020). In order to develop an affordable, robust, fast, and selective protein detection technique, DNA aptamers were evolved to recognize target proteins, and were used in Western blot. Scheme 1 summarizes the direct comparison between aptamer-based and conventional antibody-based Western blotting. Following common gel electrophoresis, transfer and blocking steps, the aptamer-based method requires only a single step of affinity detection, whereas the conventional method involves two binding steps with primary and secondary antibodies, and is typically followed by a third enzymatic chromogenic reaction. Clearly, the aptamer-based procedure would take less time and be more cost-effective, if an appropriate aptamer sequence was available.

Given that aptamers, as alternative protein affinity reagents, hold great potential in saving both the time and the cost associated with Western blotting, we first attempted to leverage previously reported aptamer sequences and to assess their performances in Western blotting. Two DNA aptamers binding thrombin (TBA) and histone protein H4 (3.13) were chosen (Supplementary Table 1) and tested for their binding capabilities to proteins immobilized on nitrocellulose membrane (Bock et al., 1992; Yu et al., 2011). Although these two aptamers have demonstrated high-affinity and high-specificity binding toward their target proteins in solution, their performances in Western blotting were far from satisfactory. TBA could not effectively recognize immobilized thrombin protein at all, while 3.13 indiscriminately labeled all four histone proteins tested on the nitrocellulose membrane (Supplementary Figure 1). We reasoned that this loss of affinity and specificity was largely due to the difference in detection conditions. In Western blotting, proteins are denatured during SDS-PAGE. And aptamers selected to recognize native proteins in solution are likely to lose their binding affinity and selectivity toward unfolded proteins immobilized on the nitrocellulose membrane.

Realizing that aptamers tend to function well in conditions where they are originally isolated, we hypothesized that an *in vitro* selection toward a target protein immobilized on the nitrocellulose membrane would likely yield aptamer sequences suitable for Western blotting. So three *in vitro* selections of DNA aptamers toward commonly used protein tags GST (27 kDa, theoretical pI 6.09), MBP (43 kDa, theoretical pI 5.46), and His-tag (theoretical pI 7.21) were devised and performed (Supplementary Figures 2, 3). GST and MBP are highly soluble protein domains which could promote solubility when fused to a protein of interest (Diguan et al., 1988; Smith and Johnson, 1988). His-tag typically consists of six consecutive histidine residues and is relatively small in size. To facilitate selection toward such a small protein tag, two different proteins, His-tagged green fluorescent protein (His-GFP) and His-tagged thioredoxin 1 (His-TrxA), were used as targets in alternate rounds of *in vitro* selection.

An ssDNA library consisting of a 40-nucleotide random region was synthesized (Supplementary Figure 4) and subject to *in vitro* selection of DNA aptamers that specifically bound target proteins immobilized on the nitrocellulose membrane. In each round of selection, the Cy5.5-labeled ssDNA library was incubated with purified target proteins that were first separated on SDS-PAGE and then transferred onto a nitrocellulose membrane. After fluorescence imaging, the band corresponding to bound DNA aptamers was excised, eluted, and amplified to initiate the next round of selection. To increase selection stringency and aptamer selectivity (Supplementary Table 2), *E. coli* cell lysate was also included in a separate lane and DNA sequences bound to cell lysate were discarded. After 18 rounds of selection, the enriched DNA pool were cloned, and sequenced.

**Figure 1:**
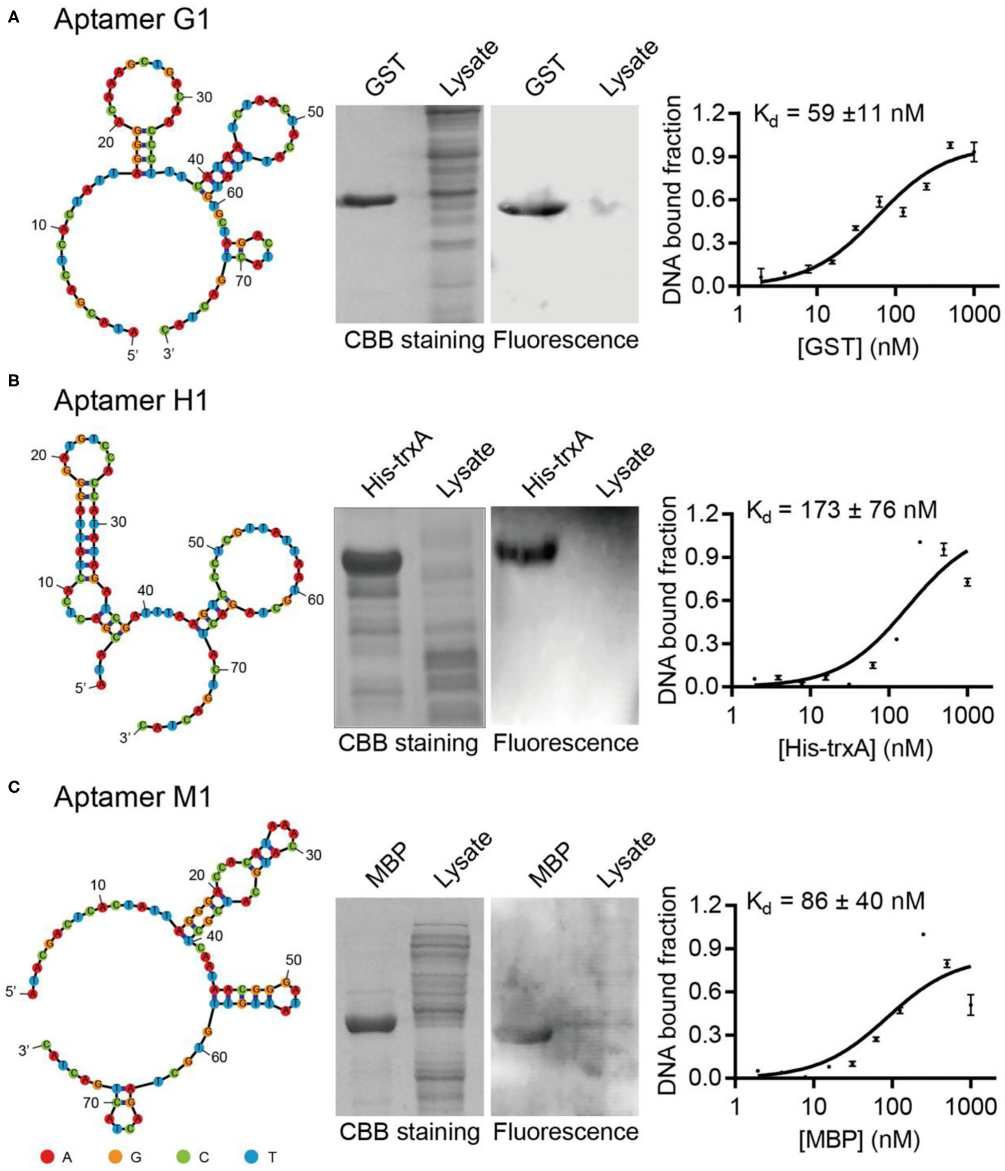
Isolated aptamers specifically recognize target proteins in Western blot. The aptamer secondary structures are predicted using Mfold and contain multiple stem-loop motifs. Purified proteins and *E. coli* cell lysates are separated on SDS-PAGE, stained with Coomassie Brilliant Blue (left) and probed with Cy5.5-labeled corresponding aptamers (right). The dissociation constants of aptamers G1, H1, and M1 toward their cognate target proteins are determined to be in the nanomolar range by dot blot. The target proteins are **(A)** GST, **(B)** His-TrxA, and **(C)** MBP, respectively. The binding curves are fitted to the data of three experimental repetitions.

Sequencing results of GST, MBP, and His-tag selections yielded 21, 36, and 30 unique DNA sequences, respectively (Supplementary Tables 3–5). All of these potential DNA aptamer sequences were chemically synthesized as Cy5.5-labeled affinity probes, and tested in Western blotting for their capabilities to specifically recognize target proteins. While some sequences failed to exhibit strong affinity or selectivity toward their target proteins, aptamers G1, H1, and M1 were able to specifically label their target proteins (Figure 1). When crude *E. coli* cell lysate was included to represent a complex protein mixture, it was clearly demonstrated that the selected aptamers bound their target proteins selectively and the observed binding was not due to non-specific adsorption. Additionally, the binding affinities for such aptamer-protein interactions were determined using dot blot assay. The dissociation constants for aptamers G1, H1, and M1 binding their target proteins were 59 nM, 173 nM, and 86 nM, respectively. Two additional aptamers (G2 and H2) showed similar binding affinity and specificity (Supplementary Figure 5), revealing that there are multiple aptamer solutions to the selective protein detection problem in a Western blot setting.

To test whether these aptamers could recognize target proteins in solution, aptamer-protein interactions were evaluated using gel shift assays (Supplementary Figures 6, 7). Interestingly, the three aptamers G1, H1, and M1 exhibited quite different behaviors toward soluble proteins in solution. Aptamer M1 maintained its binding affinity to its target MBP protein in solution, showing a clear concentration-dependent formation of aptamer-protein complex. Aptamer H1 formed complex with His-TrxA only at the highest concentration of protein. In contrast, aptamer G1 did not show appreciable binding to its target GST protein in solution. These results again support the idea that aptamer affinity is often greatly influenced by the environmental factors and aptamers tend to perform the best in conditions where they are originally selected. We also compared our selected aptamers with previously reported GST and His-tag aptamers by sequence alignment, respectively. And the results showed that there was minimal sequence homology among the sequences (Supplementary Figure 8), indicating that the G1, G2, H1, and H2 were original DNA aptamer sequences toward GST and His-tag.

**Figure 2:**
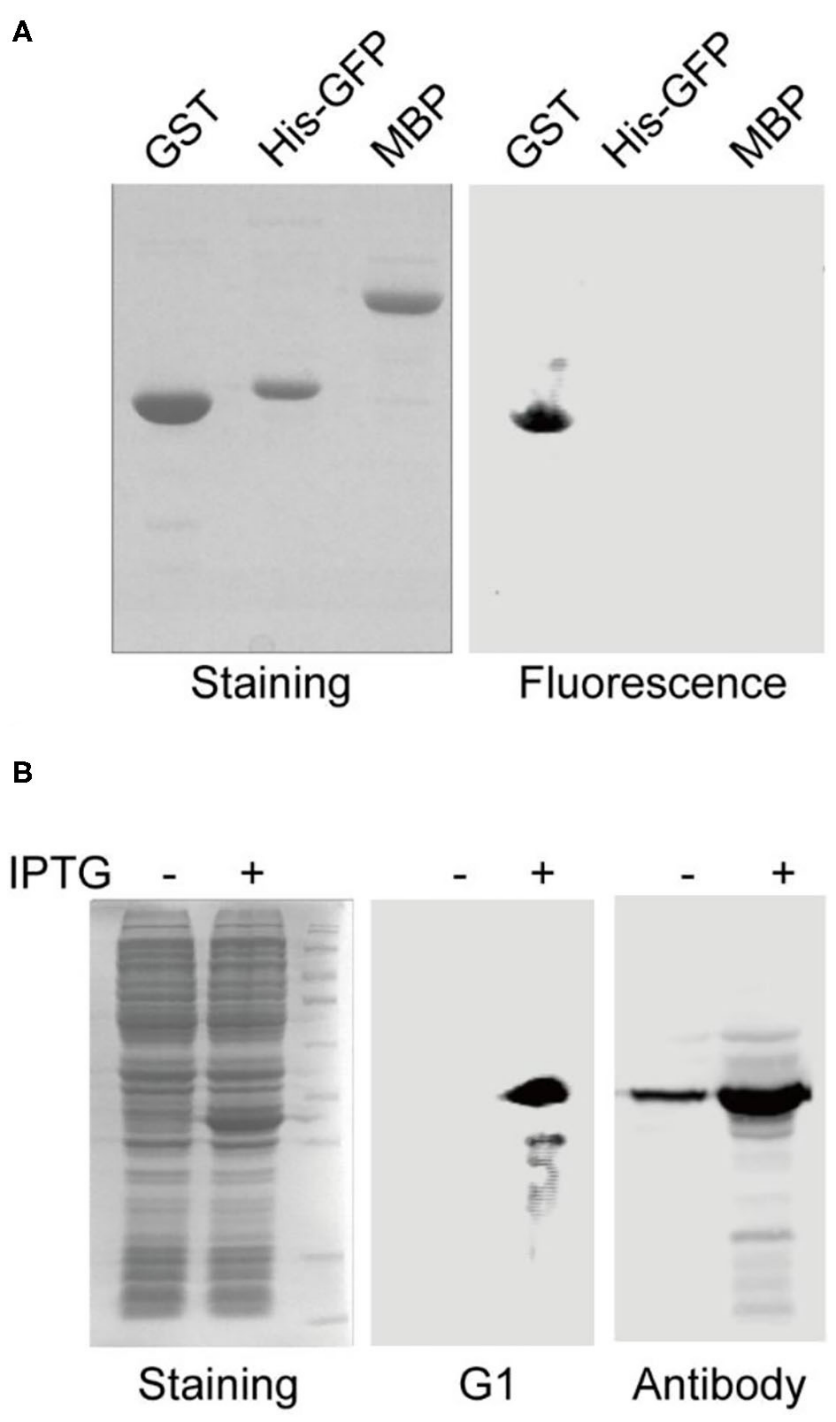
Aptamer G1 selectively label GST protein and compares favorably with antibody in Western blot. **(A)** Aptamer G1 effectively discriminates target protein GST from the other two proteins. **(B)** Aptamer G1 selectively labels GST protein in crude *E. coli* cell lysate, while anti-GST antibody shows a number of extra bands in addition to the target band.

To further validate the binding specificity of the selected aptamers, aptamer G1 was challenged to discriminate the cognate target protein (GST) from the other two off-target proteins (His-GFP and MBP). After gel electrophoresis and transfer to membrane, the fluorescence image showed that aptamer G1 selectively labeled GST protein, but not His-GFP or MBP protein (Figure 2A), indicating that the selected aptamers could effectively discriminate between cognate and non-cognate proteins. In order to directly compare the performance of this aptamer with an antibody in Western blotting, crude cell lysate of *E. coli* containing a GST-encoding plasmid before and after IPTG induction were probed with aptamer G1 and anti-GST antibody, respectively. It was shown that aptamer G1 selectively bound expressed GST protein in crude cell lysate. In contrast, the antibody not only labeled the target protein, but also showed a number of non-specific bands (Figure 2B). These results suggested that using such selected DNA aptamers could achieve more selective protein detection in Western blotting in a cost-effective and time-saving fashion.

**Figure 3:**
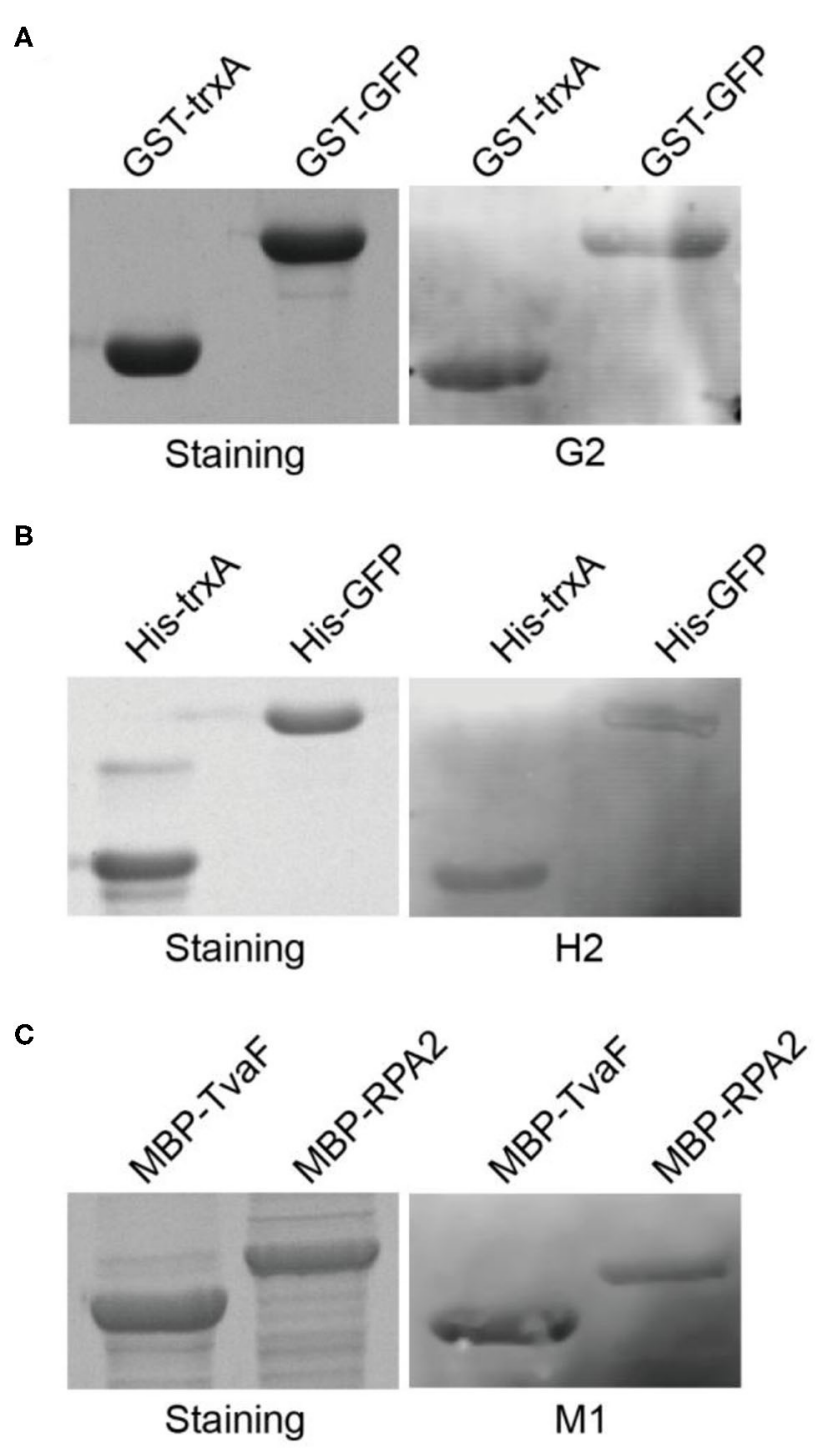
Selected aptamers are able to label fusion proteins in Western blot. **(A)** Aptamer G2 recognizes both TrxA and GFP proteins with GST tags. **(B)** Aptamer H2 recognizes his-tagged TrxA and GFP proteins. **(C)** Aptamer M1 recognizes both TvaF and RPA2 proteins with MBP tags.

These three target proteins (GST, MBP, and His-tag) chosen in this study were commonly used affinity tags in protein purification and detection. In order to take full advantage of the selected aptamers, we next asked whether the selected aptamers could recognize and label fusion proteins with corresponding tags. Two fusion proteins with GST tags (GST-TrxA and GST-GFP) were expressed and purified. Following SDS-PAGE and transfer to nitrocellulose membrane, aptamer G2 clearly labeled both two fusion proteins (Figure 3A). Similarly, his-tagged fusion proteins (His-TrxA and His-GFP) and MBP-tagged fusion proteins (MBP-TvaF and MBP-RPA2) were also effectively detected by aptamer H2 and M1, respectively (Figures 3B,C). These results highlighted the successful use of DNA aptamers in fusion protein detection, and suggested that DNA aptamers could be developed as a robust, simple, and affordable alternative affinity reagent for protein detection in Western blotting.

## Discussion

Conventional Western blotting relies on antibodies for specific protein recognition, but high-quality antibodies are not always available, especially when the protein of interest is less immunogenic or even toxic. In contrast, aptamers can be generated to bind essentially any protein target, including proteins with subtle changes like post-translational modifications (Williams et al., 2009). In addition, an aptamer can be easily synthesized and modified, and annealed to restore its function after thermal denaturation. All of these advantageous features make aptamers an attractive alternative as protein affinity reagent.

A typical Western blotting procedure requires two incubation steps with primary and secondary antibodies followed by a color development reaction. Clearly, this procedure would be simplified and more cost- and time-effective if a fluorescently labeled aptamer was used in place of two antibodies. Previous efforts replaced the primary antibody with biotin- or digoxigenin-labeled aptamer, but still required further incubation with streptavidin or anti-digoxigenin antibody conjugated with horseradish peroxidase and a following color development reaction (Martin et al., 2013; Li et al., 2017). To take the full advantage of the ease of aptamer production and modification, in this work we used fluorescently labeled aptamer to directly visualize proteins on nitrocellulose membrane. Such aptamer-based protein detection technique greatly reduced time as well as cost.

Aptamers have found broad applications in fields such as analytical chemistry, chemical biology, and molecular medicine. However, the performance of a particular aptamer sequence is often heavily influenced by its working environment. Of these, target immobilization is an important factor. Early *in vitro* selections were often directed toward target molecules immobilized on a solid support, which facilitated separation of active aptamer sequences. Soon it was realized that target immobilization strategy inevitably eliminated potential binding sites for aptamers and also resulted in a kinetic bias against the best binders. Therefore, selection methods were developed that allowed binding to occur free in solution (Mendonsa and Bowser, 2004). However, aptamers identified using this technique do not necessarily operate well when target proteins are attached to a stationary support like in ELISA and SPR assays. As shown in this study, two aptamers (TBA and 3.13) that were originally selected to recognize proteins immobilized on affinity column and free in solution were not able to selectively label the cognate targets. These results indicated that the actual working conditions should always be taken into consideration when designing an *in vitro* selection strategy of aptamers.

## Conclusion

In this study, multiple DNA aptamers were generated by *in vitro* selection that selectively recognized GST, MBP, and His-tag proteins on the nitrocellulose membrane, respectively. These aptamers exhibited specific binding to their target proteins with nanomolar affinities. Direct comparison of aptamer-based with conventional antibody-based Western blotting demonstrated that aptamer labeling required less time and gave a cleaner background. Lastly, the identified aptamer sequences were also effective in recognition of fusion proteins, which greatly expands the scope of potential applications of such aptamers. This work suggested that the methodology developed here could be extended to *in vitro* selections toward other tag proteins, and the aptamers generated from such selections would represent a type of simpler and more affordable alternative affinity reagent for protein immunoblotting analysis.

## Data Availability Statement

All datasets generated for this study are included in the article/Supplementary Material.

## Author Contributions

HY conceived the project. YW performed the experiment. ZL and HY wrote the manuscript with input from all authors. All authors discussed the results and commented on the manuscript.

## Conflict of Interest

The authors declare that the research was conducted in the absence of any commercial or financial relationships that could be construed as a potential conflict of interest.
