# Supplementary material for: Aptamer-Based Western Blot for Selective Protein Recognition
Source: Front Chem. 2020 Oct 29;8:570528. doi: 10.3389/fchem.2020.570528 (PMC7658645; doi:10.3389/fchem.2020.570528)
Supplement: Supplementary file 1 [file Table_1.docx]

Supplementary Material

**Aptamer-based Western blot for selective protein recognition**

Yao Wang ^1,2,3^, Zhe Li^1,4^, and Hanyang Yu^*1,2,3^

1 Department of Biomedical Engineering, College of Engineering and Applied Sciences, Jiangsu Key Laboratory of Artificial Functional Materials, Nanjing University, Nanjing, Jiangsu 210023, China

2 State Key Laboratory of Coordination Chemistry, Nanjing University, Nanjing, Jiangsu 210023, China

3 Chemistry and Biomedicine Innovation Center (ChemBIC), Nanjing University, Nanjing, Jiangsu 210023, China

4 State Key Laboratory of Analytical Chemistry for Life Science, Nanjing University, Nanjing, Jiangsu 210023, China

* Corresponding author

E-mail address: hanyangyu@nju.edu.cn

**
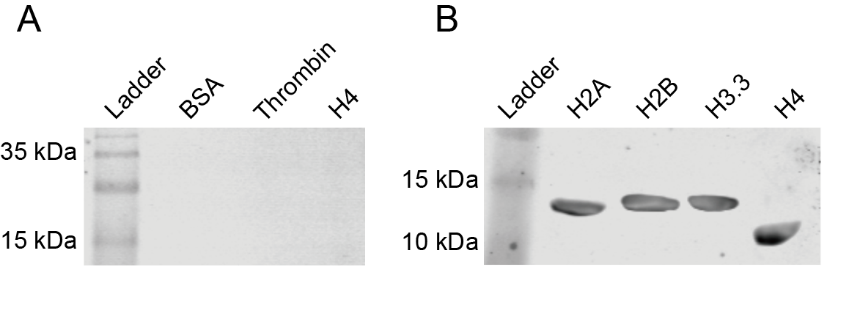
**

**Figure S1.** Aptamers previously selected towards target proteins in solution could not recognize denatured proteins on nitrocellulose membrane effectively and selectively. (A) Thrombin binding aptamer (TBA) could not bind cognate or noncognate proteins on nitrocellulose membrane. (B) Histone H4 aptamer 3.13 bound all four types of histone proteins with no selectivity.

**
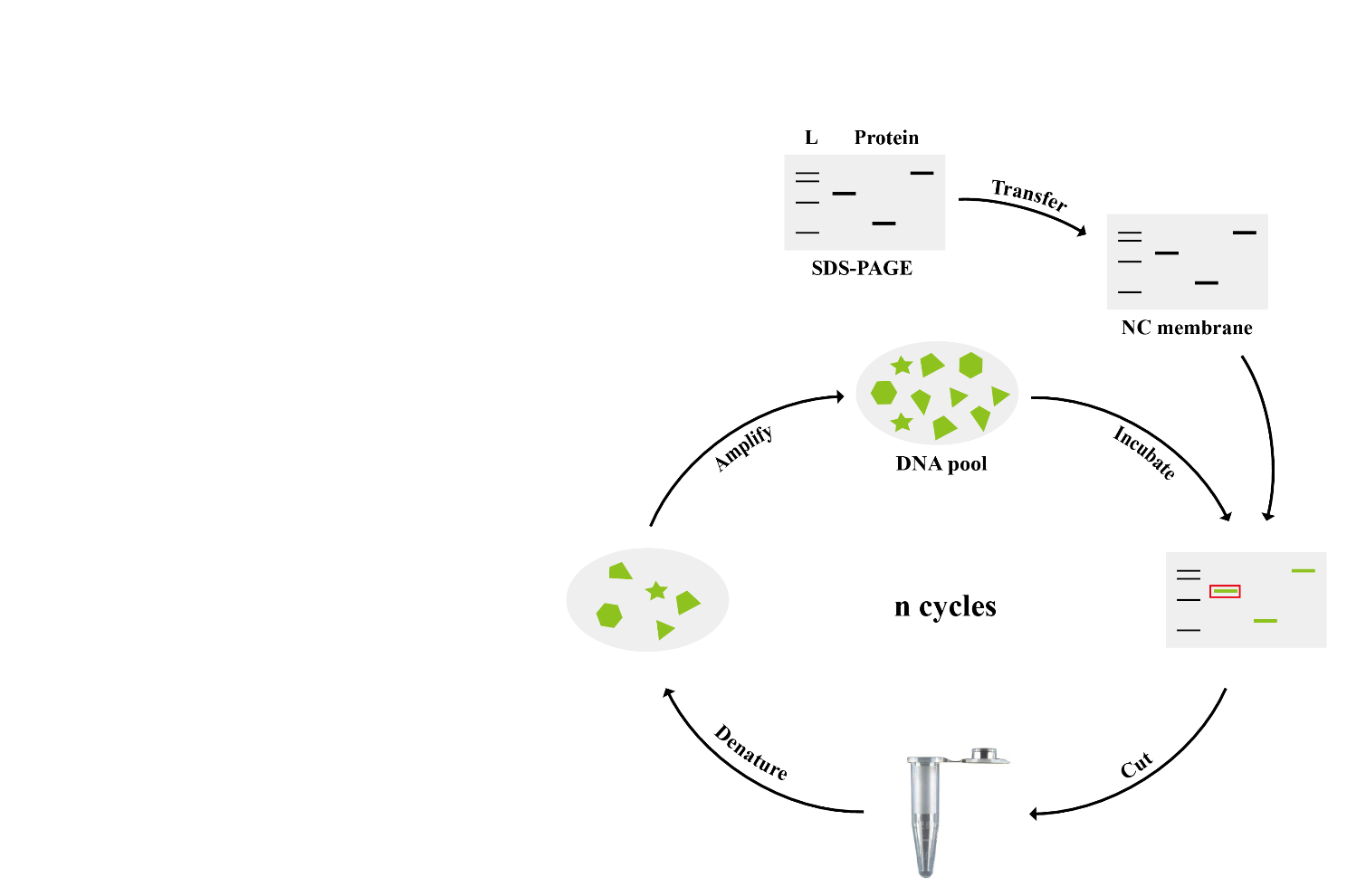
**

**Figure S2**. In vitro selection scheme of DNA aptamers for selective protein recognition in Western blot. Target proteins were first separated on SDS-PAGE and then transferred to nitrocellulose (NC) membrane. The DNA pool was incubated with target proteins immobilized on nitrocellulose membrane, and bound DNA sequences were collected and amplified to initiate the next round of selection.


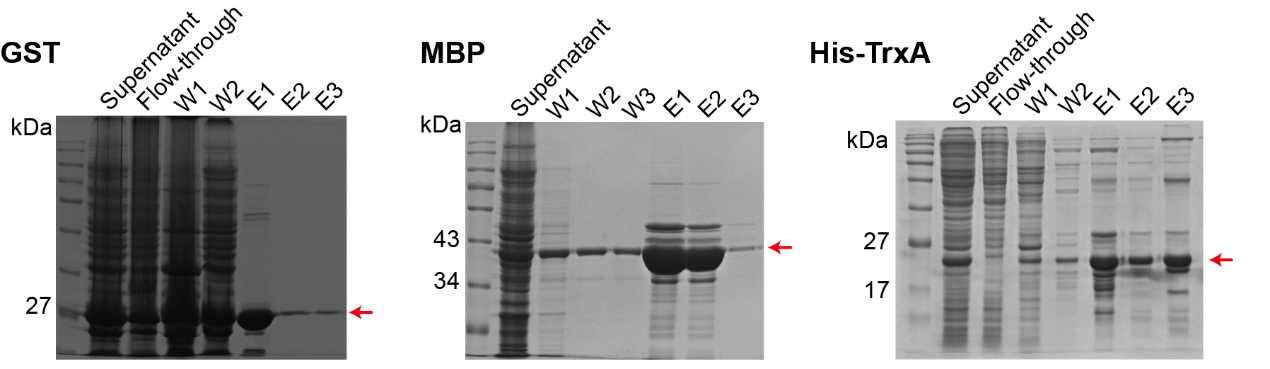


**Figure S3**. Expression and purification of three tag proteins. Three target proteins were induced to express in *E. coli* and products at different purification stages were analyzed on SDS-PAGE. W: products recovered from affinity column using wash buffer. E: products recovered from affinity column using elution buffer. Red arrows indicate target proteins.


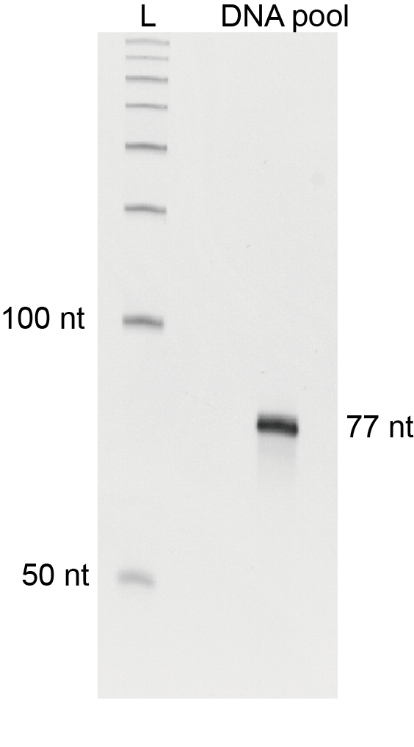


**Figure S4**. PAGE analysis of chemically synthesized initial DNA library.


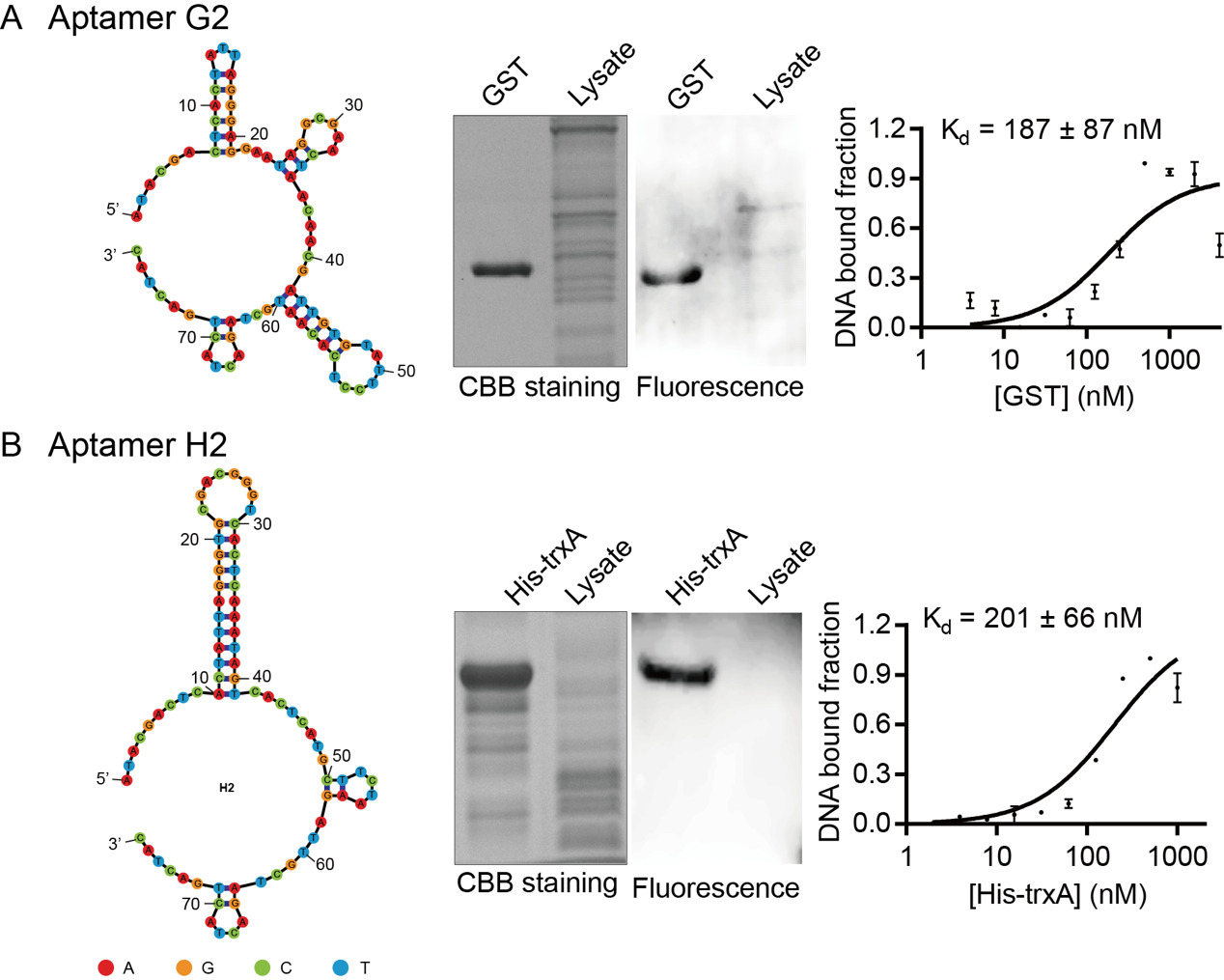


**Figure S5**. Aptamers G2 and H2 selectively bound target proteins on nitrocellulose membrane with nanomolar affinities. The Mfold-predicted secondary structures of aptamers were shown. Purified proteins and *E. coli* cell lysates were stained with Coomassie Brilliant Blue (left) and probed with Cy5.5-labeled corresponding DNA aptamers (right). Affinity curves were fitted to the data of three experimental repeats. (A) Aptamer G2 bound to GST protein. (B) Aptamer H2 bound to His-TrxA protein.


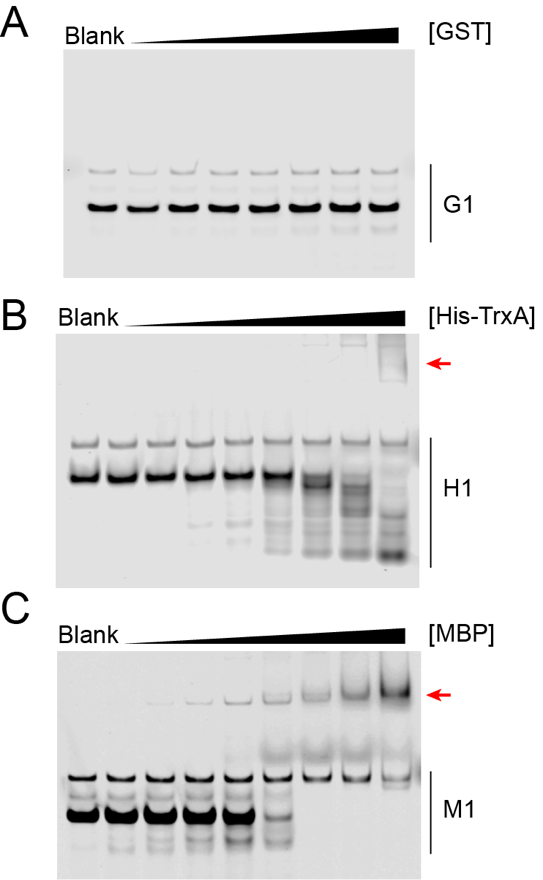


**Figure S6.** Gel shift analysis of aptamers binding to target proteins in solution. Aptamers (50 nM) were allowed to interact with target proteins of different concentrations in solution, and the samples were then analyzed by native gel electrophoresis. (A) Aptamer G1 and GST protein; (B) Aptamer H1 and His-TrxA protein; (C) Aptamer M1 and MBP protein. The red arrows indicate the aptamer-protein complex. Blank: aptamer alone. The multiple bands in the blank lane might reflect that the aptamers could fold into several distinct tertiary structures of different mobilities.


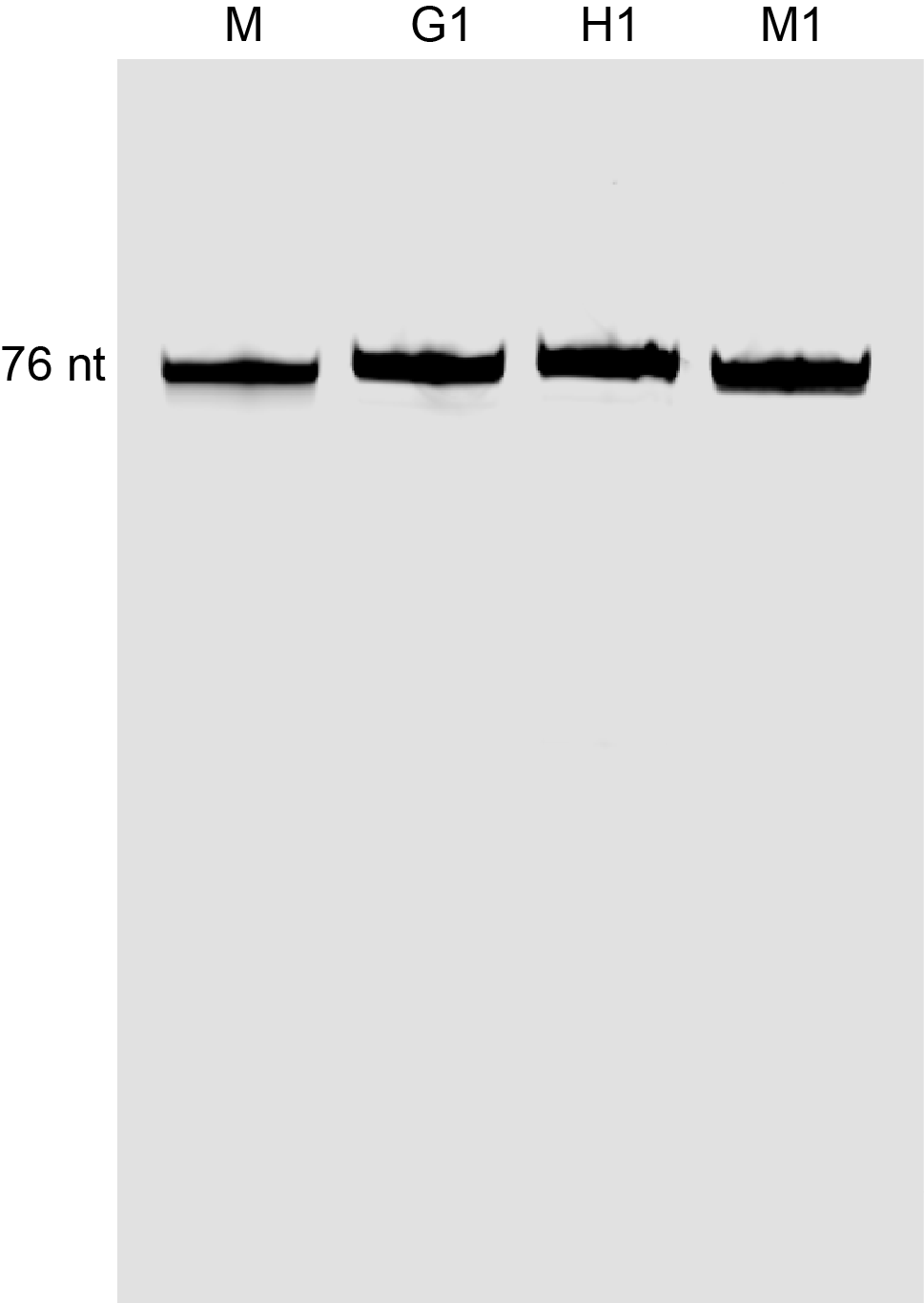


**Figure S7.** Purity analysis of aptamers G1, H1 and M1 by denaturing gel electrophoresis. Since multiple bands of aptamers were observed during native gel electrophoresis, the purities of the aptamers were determined on a denaturing gel. All three aptamers appear as a single band of the correct length.

**
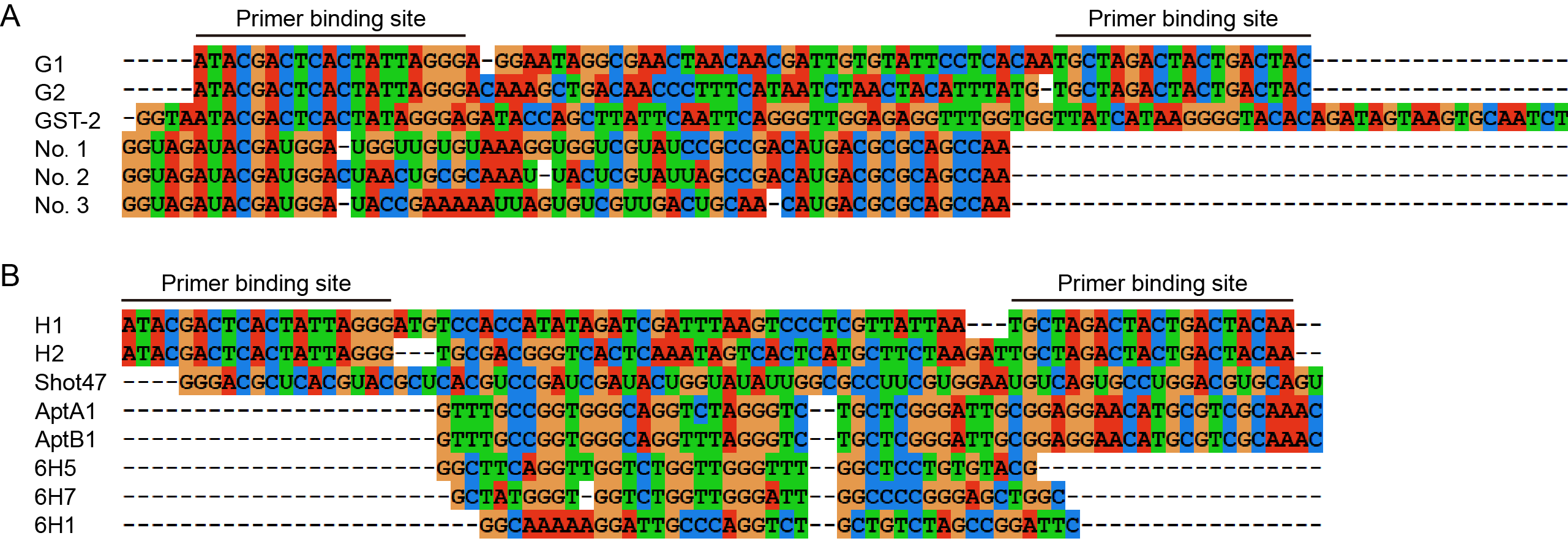
**

**Figure S8.** Sequence alignment of GST and His-tag aptamers with previously reported aptamer sequences. (A) Aptamers G1 and G2 showed minimal sequence homology to the previously reported GST aptamers. G1 and G2 are two aptamer sequences identified in this study. GST-2 was reported by Sekhon *et al*. (Sekhon et al., 2017). Sequences no. 1-3 were reported by Yoshida *et al*. (Yoshida et al., 2010). (B) Aptamers H1 and H2 also showed minimal sequence homology to the previously reported His-tag aptamers. H1 and H2 are two aptamer sequences identified in this study. Shot47 was reported by Tsuji *et al*. (Tsuji et al., 2009). AptA1 and AptB1 were reported by Bartnicki *et al*. (Bartnicki et al., 2016). 6H5, 6H7 and 6H1 were reported by Doyle *et al*. (Doyle et al., 2008).

**Table S1**. DNA sequences used in this study.

| Name | Sequence (5’-3’) |
| --- | --- |
| 3.13 | GAGTGTGGTTCCCGGGAGGGCGCCTACGGGTCCCGTATTCGGATTTGTGC |
| TBA | GGTTGGTGTGGTTGG |
| ssDNA library | ATACGACTCACTATTAGGG-N_40_-TGCTAGACTACTGACTAC |
| Forward primer | Cy5.5-ATACGACTCACTATTAGGG |
| Reverse primer | Biotin-GTAGTCAGTAGTCTAGCA |
| G1 | ATACGACTCACTATTAGGGACAAAGCTGACAACCCTTTCATAATCTAACTACATTTATGTGCTAGACTACTGACTAC |
| G2 | ATACGACTCACTATTAGGGAGGAATAGGCGAACTAACAACGATTGTGTATTCCTCACAATGCTAGACTACTGACTAC |
| H1 | ATACGACTCACTATTAGGGATGTCCACCATATAGATCGATTTAAGTCCCTCGTTATTAATGCTAGACTACTGACTACAA |
| H2 | ATACGACTCACTATTAGGGTGCGACGGGTCACTCAAATAGTCACTCATGCTTCTAAGATTGCTAGACTACTGACTACAA |
| M1 | ATACGACTCACTATTAGGGACCACATAAACATGCATCGCTCAATAACGGGATATTGTTGTGCTAGACTACTGACTACAA |

**Table S2.** Conditions for each round of *in vitro* selection.

| Round | Target protein conc. (mg/mL) | Blocking RNA/DNA conc. (μg/mL) | NaCl conc. in washing step (mM) | Washing time |
| --- | --- | --- | --- | --- |
| 1 | 3.5 | 0 | 150 | 4*5 min |
| 2 | 3.0 | 10 | 150 | 4*5 min |
| 3 | 2.5 | 10 | 150 | 4*5 min |
| 4 | 2.0 | 10 | 150 | 4*5 min |
| 5 | 1.5 | 10 | 150 | 4*5 min |
| 6 | 1.0 | 10 | 150 | 4*5 min |
| 7 | 1.0 | 50 | 500 | 4*5 min |
| 8 | 1.0 | 100 | 500 | 4*5 min |
| 9 | 1.0 | 200 | 500 | 4*5 min |
| 10 | 1.0 | 500 | 500 | 4*5 min |
| 11 | 1.0 | 500 | 500 | 4*10 min |
| 12 | 1.0 | 500 | 500 | 4*10 min |
| 13 | 1.0 | 500 | 500 | 4*10 min |
| 14 | 1.0 | 500 | 500 | 6*10 min |
| 15 | 1.0 | 500 | 500 | 6*10 min |
| 16 | 1.0 | 500 | 500 | 6*10 min |
| 17 | 1.0 | 500 | 500 | 6*10 min |
| 18 | 1.0 | 500 | 500 | 6*10 min |

**Table S3.** Sequencing results of GST aptamer selection output. Primer-binding sites are underlined.

| Name | Sequence (5’-3’) |
| --- | --- |
| **G1** | ATACGACTCACTATTAGGG ACAAAGCTGACAACCCTTTCATAATCTAACTACATTTATG TGCTAGACTACTGACTAC |
| **G2** | ATACGACTCACTATTAGGG AGGAATAGGCGAACTAACAACGATTGTGTATTCCTCACAA TGCTAGACTACTGACTAC |
| G3 | ATACGACTCACTATTAGGG AGCTTGAAGAAGTTGAAGCCTACCTTTTTACCGTTTTCCG TGCTAGACTACTGACTAC |
| G4 | ATACGACTCACTATTAGGG AAGTACTTACCAAGCAACAAAATTCTTCAACGCTTCGTTG TGCTAGACTACTGACTAC |
| G5 | TTACGACTCACTATTAGGG TAAGCAAATTGGACAACCTAAAGGACTCACTTTGCCATTG TGCTAGACTACTGACTAC |
| G6 | ATACGACTCACTATTAGGG TACTCGCGGACTTTACTCAATCATAGCCAGCACATTCAAT TGCTAGACTACTGACTAC |
| G7 | ATACGACTCACTATTAGGG GCGTTACGCACAAGTCTCTATAATTTCCGTATAAACTAGT TGCTAGACTACTGACTAC |
| G8 | ATACGACTCACTATTAGGG CGACTCAAGGTCTTATCTGCACCAAAAAAACCCCTCTGCA TGCTAGACTACTGACTAC |
| G9 | ATACGACTCACTATTAGGG CAGGACGCAAAACGAATCGTGTCCTAGGCCTTAACCCTTG TGCTAGACTACTGACTAC |
| G10 | ATACGACTCACTATTAGGG CCACGACGCGTGTTAATTCATTCGACTCAACACCTTGCCA TGCTAGACTACTGACTAC |
| G11 | ATACGACTCACTATTAGGG CTCAGTCCTGACACCAACACGGATGCACAGATCTTTACCT TGCTAGACTACTGACTAA |
| G12 | ATACGACTCACTATTAGGG ATTCATGACCCACAGACGACGTCCAATTACGTCCTTGTAA TGCTAGACTACTGACTAC |
| G13 | ATACGACTCACTATTAGGG AAAAGATAACTTCACACGACTGCATCCCAGCGTAGCAAGT TGCTAGACTACTGACTAC |
| G14 | ATACGACTCACTATTAGGG AACACCAATCACCATACTGGTACCGAACGTACCCTTGAGC TGCTAGACTACTGACTAC |
| G15 | ATACGACTCACTATTAGGG CTTGTGAACAAAACGCCTATAAGCCTTACCATATGTCCCG TGCTAGACTACTGACTAC |
| G16 | ATACGACTCACTATTAGGG TCCATACGTGGTCAAATCGATAACGCAAGACAT TGCTAGACTACTGACTAC |
| G17 | ATACGACTCACTATTAGGG GCTCCAGTCCAACGCAGACCCTCACACAGCCGGTTCTCCA TGCTAGACTACTGACTAC |
| G18 | ATACGACTCACTATTAGGG GGACGAACGTACACCACTTCGTCTTTGCTATCTCCCTCGA TGCTAGACTACTGACTAC |
| G19 | ATACGACTCACTATTAGGG TGACAGCTTCAACCTTACTGTCTCATACATTCCTTTAATT TGCTAGACTACTGACTAC |
| G20 | ATACGACTCACTATTAGGG CATAAGCAAGCAACGATCCACCACACAGTTCGCTCTGCAA TGCTAGACTACTGACTAC |
| G21 | ATACGACTCACTATTAGGG CAACACAAGGACACACAATTATTATTGCTGTTCCTTTACT TGCTAGACTACTGACTAC |

| Name | Sequence (5’-3’) |
| --- | --- |
| **H1** | ATACGACTCACTATTAGGG ATGTCCACCATATAGATCGATTTAAGTCCCTCGTTATTAA TGCTAGACTACTGACTACAA |
| **H2** | ATACGACTCACTATTAGGG TGCGACGGGTCACTCAAATAGTCACTCATGCTTCTAAGAT TGCTAGACTACTGACTACAA |
| H3 | ATACGACTCACTATTAGGG CTGAGACCCGAATCTCAGTTCTACCACCGTCTATTATGCA TGCTAGACTACTGACTACAA |
| H4 | ATACGACTCACTATTAGGG TCTCAAGCGCCGTTGAAAACATTTATACCAACTCGGTTTT TGCTAGACTACTGACTACAA |
| H5 | ATACGACTCACTATTAGGG TTCCTGCATAACTCTAGCATGGATCACAACTTTCTTCATA TGCTAGACTACTGACTACAA |
| H6 | ATACGACTCACTATTAGGG TGACACGGTGAGTAAGAACTCCTACTCTATCTCCGTTACA TGCTAGACTACTGACTACAA |
| H7 | ATACGACTCACTATTAGGG GATGTACCTAGTTACCCAACTCTTCACTAACGTCATCTCG TGCTAGACTACTGACTACAA |
| H8 | ATACGACTCACTATTAGGG CTGGATATAGCTGCCCTAGACTATGAAGTTTTCTTTCCTA TGCTAGACTACTGACTACAA |
| H9 | ATACGACTCACTATTAGGG TACAAGAACGAAACTACCAGCGTATTCTCTCATAGTATTC TGCTAGACTACTGACTACAA |
| H10 | ATACGACTCACTATTAGGG CTCAACCACATCTGTCATCGTTAACTATATGCGAATAGTA TGCTAGACTACTGACTACAA |
| H11 | ATACGACTCACTATTAGGG AAACGCCCACAATATAAAACAGGGACGTTGTTAATCTACT TGCTAGACTACTGACTACAA |
| H12 | ATACGACTCACTATTAGGG TCACACCAGATTGTAATACTACCAGTTCACAAGCGCCGAT TGCTAGACTACTGACTACAA |
| H13 | ATACGACTCACTATTAGGG CGACCCGTTCAGAGCAACTATCGCCGTTCTTTGCCCCTGT TGCTAGACTACTGACTACAA |
| H14 | ATACGACTCACTATTAGGG AATGGGTAGAATTGCTGAGTTCGCCCGACCAATATAAGTT TGCTAGACTACTGACTACAA |
| H15 | ATACGACTCACTATTAGGG TCCTTGAAGTCTACGCCTTCTCCACGTCCAGTTTCCATAC TGCTAGACTACTGACTACAA |
| H16 | ATACGACTCACTATTAGGG CAATCGAGCAACCTCGGTCACCCTTGTATCGCAGACTCCC TGCTAGACTACTGACTACAA |
| H17 | ATACGACTCACTATTAGGG ACCGGACTGGTTTCTACATACTGGCCAATACATCAGTTC TGCTAGACTACTGACTACAA |
| H18 | ATACGACTCACTATTAGGG CGCGCACGATGCCAAACCGCGAAAATTTCAATTGTTGTTT TGCTAGACTACTGACTACAA |
| H19 | ATACGACTCACTATTAGGG AACTTTCGATCTGTGACATTCCACTTAGATTGCAA TGCTAGACTACTGACTACAA |
| H20 | ATACGACTCACTATTAGGG ATGCACAATTCACCATTCGTAAAGTGATACATCCACTTGG TGCTAGACTACTGACTACAA |
| H21 | ATACGACTCACTATTAGGG ATAGTCTCGCAAATTACATGCCTTTCGACACGAACCGTCA TGCTAGACTACTGACTACAA |
| H22 | ATACGACTCACTATTAGGG ACGCAACCTGGCCATGCCACGCCTCACTACGTGTTCATAG TGCTAGACTACTGACTACAA |
| H23 | ATACGACTCACTATTAGGG TCAGTCAGTATAGACCTCCACCCGACTTCTCACCTTGATA TGCTAGACTACTGACTACAA |
| H24 | ATACGACTCACTATTAGGG TTATATACCATGACGCCTCCGCATCCAAATATTGGTTCTA TGCTAGACTACTGACTACAA |
| H25 | ATACGACTCACTATTAGGG CCTCGAAGAGCACCGTACATTTCCGAAAGTTCTTTCTTAG TGCTAGACTACTGACTACAA |
| H26 | ATACGACTCACTATTAGGG ATAACGATGAATCTGCGGTTCGCCCTATACCGTCTTTTCT TGCTAGACTACTGACTACAA |
| H27 | ATACGACTCACTATTAGGG TGACCACATCTTATCTAGGTAAGTCTAATTCAGTACCCT TGCTAGACTACTGACTACAA |
| H28 | ATACGACTCACTATTAGGG TATCAAAACTTGCTCGAGTATCGGACCGGTCCCTCACTTG TGCTAGACTACTGACTACAA |
| H29 | ATACGACTCACTATTAGGG ATAAAACGTCATACAGATGTACATCTTGCTGCATGGGTGT TGCTAGACTACTGACTACAA |
| H30 | ATACGACTCACTATTAGGG TAGGTCAGATACGAGACATTGAGTTCTCCTTTCTTACGTA TGCTAGACTACTGACTACAA |

**Table S4**. Sequencing results of His-tag aptamer selection output. Primer-binding sites are underlined.

| Name | Sequence (5’-3’) |
| --- | --- |
| **M1** | ATACGACTCACTATTAGGG ACCACATAAACATGCATCGCTCAATAACGGGATATTGTTG TGCTAGACTACTGACTAC |
| M2 | ATACGACTCACTATTAGGG AATAGCCTAGGTATGTAACTCATCGTATGTGGTTCAAAAA TGCTAGACTACTGACTAC |
| M3 | ATACGACTCACTATTAGGG CTGTTGTCCAGGACTACGCACCGATCAGCGTTCCCGTTAC TGCTAGACTACTGACTAC |
| M4 | ATACGACTCACTATTAGGG AATGCACATCATATGCCATCCTCCTATTAGTCCGCCCATA TGCTAGACTACTGACTAC |
| M5 | ATACGACTCACTATTAGGG TTAAACCGTATGGCAATATTACACTGCATTATACTTAGCT TGCTAGACTACTGACTAC |
| M6 | ATACGACTCACTATTAGGG GCTAACACTGGGAGCAAGTAAAGCAAATCTTCTTTTCTTG TGCTAGACTACTGACTAC |
| M7 | ATACGACTCACTATTAGGG TCTTACAAGCATTTGCGCACGGACTTCTCGTCATCCGAAC TGCTAGACTACTGACTAC |
| M8 | ATACGACTCACTATTAGGG TGACTTACTCGAGACGGTGACTGTGCTCTAATTACGAATC TGCTAGACTACTGACTAC |
| M9 | ATACGACTCACTATTAGGG AAGCAACAATGTAGCCTTCTAACCAGGTATTTGGTTACTT TGCTAGACTACTGACTAC |
| M10 | ATACGACTCACTATTAGGG CACGAATCGCTCAATTTCAAAGGTCAATGCGGATAGTTAA TGCTAGACTACTGACTAC |
| M11 | ATACGACTCACTATTAGGG GAAACTCGTCAATTCGGACCGTGCTTCGTCCTCCGATGTA TGCTAGACTACTGACTAC |
| M12 | ATACGACTCACTATTAGGG ACCAACTACAGCATATCTGATTACACATCTCCCACAAGCT TGCTAGACTACTGACTAC |
| M13 | ATACGACTCACTATTAGGG TAGCTATCACCTTCCGCACAACAGGAATCTTTGCACCAAA TGCTAGACTACTGACTAC |
| M14 | ATACGACTCACTATTAGGG TCCTACCAGGCAACTGCAATGAACGAATTTCATTGATCTT TGCTAGACTACTGACTAC |
| M15 | ATACGACTCACTATTAGGG TTTAACCGCCATCCTATAGAATGGTTACTACTTATCACAC TGCTAGACTACTGACTAC |
| M16 | ATACGACTCACTATTAGGG TATCTCAAGCTTCAACATTCATGACCCCGTCAATATATTA TGCTAGACTACTGACTAC |
| M17 | ATACGACTCACTATTAGGG TCCTCGCAGCAACCACCCTAACAACAGTCTGTACCTGTTA TGCTAGACTACTGACTAC |
| M18 | ATACGACTCACTATTAGGG CACATGACTAGACTAATTATTATTGCATCTTAGGCTTCCT TGCTAGACTACTGACTAC |
| M19 | ATACGACTCACTATTAGGG GACCTTGGTAATACCCTAGTACTCCCTCAGTTTCCTTCTG TGCTAGACTACTGACTAC |
| M20 | ATACGACTCACTATTAGGG GTACGGCTCATCATCGGTCATAATTTCCCATTTATACCT TGCTAGACTACTGACTAC |
| M21 | ATACGACTCACTATTAGGG CACAAAGACCATACCATATTAGAAGTTACTTCCACTCTCG TGCTAGACTACTGACTAC |
| M22 | ATACGACTCACTATTAGGG GACGCCAGTCCACAACCCCCACGGTGATCCTTACTTTTA TGCTAGACTACTGACTAC |
| M23 | ----GACTCACTATTAGGG GAAGCCCTTTTAACTATCGGTTCTGACCGAAATGGTCAAA TGCTAGACTACTGACTAC |
| M24 | ATACGACTCACTATTAGGG CCATATAAGCTCACGCCCCACGAATACGCCCTTTTCTTCG TGCTAGACTACTGACTAC |
| M25 | ATACGACTCACTATTAGG- TAACAGCCACACTCTCGACTTAAAAACCGGCAGTACTATT TGCTAGACTACTGACTAC |
| M26 | ATACGACTCACTATTAGGG CTGAGCCAAAATTCCAGTACCTTGATCCCCATTTTTGCCA TGCTAGACTACTGACTAC |
| M27 | ATACGACTCACTATTAGG- TACAGCAATGCCACATTCCTCTTACTAACGATCGCTTAGC TGCTAGACTACTGACTAC |
| M28 | ATACGACTCACTATTAGGG ATACTGTACATCAACATTACCCCCTCATTGCCTTGCTTCG TGCTAGACTACTGACTAC |
| M29 | ATACGACTCACTATTAGGG GTAAACGGACCAAGTATCAACAGTTTTCAGTCCGATCACA TGCTAGACTACTGACTAC |
| M30 | ATACGACTCACTATTAGGG TATGGCTCGAAAACACGCCCGAGACACAGGTCCACGACAG TGCTAGACTACTGACTAC |
| M31 | ----GACTCACTATTAGGG TCAACACTTTCACTCGTACCACTACTAAGAGTTCAACG TGCTAGACTACTGACTAC |
| M32 | ATACGACTCACTATTAGGG ATGAAAAGCCTAAAAATCCGGCCAGCGTGCCACTTCCATG TGCTAGACTACTGACTAC |
| M33 | ATACGACTCACTATTAGGG TACTGCCCAGATAACTATGCACTTGCATCATACGTTGGCT TGCTAGACTACTGACTAC |
| M34 | ATACGACTCACTATTAGGG TGTAGCGGCTTTCCCTTAAACCCATTTAATATCTTCAGGA TGCTAGACTACTGACTAC |
| M35 | ATACGACTCACTATTAGGG ACTCCTCGCGATTCAACCGAACACGGGCATATAACTC TGCTAGACTACTGACTAC |
| M36 | ATACGACTCACTATTAGGG CAAGTTACAACTCTGCCTATGGATCCGGCTTATTCATTT TGCTAGACTACTGACTAC |

**Table S5**. Sequencing results of MBP aptamer selection output. Primer-binding sites are underlined.

**References**

Bartnicki, F., Kowalska, E., Pels, K., and Strzalka, W. (2016). DNA aptamers binding the histidine tag and their application. United states patent application 14/780635.

Doyle, S.A., Creek, W., Murphy, M.B., and Park, S. (2008). Aptamers and methods for their in vitro selection and uses thereof. United states patent application 10/934856. 2008. 02.12.

Sekhon, S.S., Um, H.J., Shin, W.R., Lee, S.H., Min, J., Ahn, J.Y., et al. (2017). Aptabody-aptatope interactions in aptablotting assays. *Nanoscale* 9**,** 7464-7475. doi: 10.1039/c7nr01827d.

Tsuji, S., Tanaka, T., Hirabayashi, N., Kato, S., Akitomi, J., Egashira, H., et al. (2009). RNA aptamer binding to polyhistidine-tag. *Biochem Biophys Res Commun* 386**,** 227-231. doi: 10.1016/j.bbrc.2009.06.014.

Yoshida, Y., Penmetca, K.K.R., Nishikawa, S., and Waga, I. (2010). High-affinity RNA aptamer molecule against glutathione-s-transferase protein. United States patent application 11/887431. 2010. 02. 11.
